# Latent Profile Analysis to Survey Positive Mental Health and Well-Being: A Pilot Investigation Insight Tunisian Facebook Users

**DOI:** 10.3389/fpsyt.2022.824134

**Published:** 2022-04-07

**Authors:** Noomen Guelmami, Amayra Tannoubi, Nasr Chalghaf, Mouna Saidane, Jude Kong, Luca Puce, Azaiez Fairouz, Nicola Luigi Bragazzi, Roobaea Alroobaea

**Affiliations:** ^1^Postgraduate School of Public Health, Department of Health Sciences (DISSAL), University of Genoa, Genoa, Italy; ^2^Group for the Study of Development and Social Environment (GEDES), Faculty of Human and Social Science of Tunis, Tunis, Tunisia; ^3^Department of Human and Social Sciences, Higher Institute of Sport and Physical Education of Kef, University of Jendouba, Jendouba, Tunisia; ^4^Department of Human Sciences, Higher Institute of Sport and Physical Education of Sfax, University of Sfax, Sfax, Tunisia; ^5^Department of Neuroscience, University of Genoa, Genoa, Italy; ^6^Laboratory for Industrial and Applied Mathematics (LIAM), York University, Toronto, ON, Canada; ^7^Department of Mathematics and Statistics, York University, Toronto, ON, Canada; ^8^Department of Computer Science, College of Computers and Information Technology, Taif University, Taif, Saudi Arabia

**Keywords:** latent profile, survey, positive psychology, mental health, COVID-19, Facebook

## Abstract

**Background:**

To examine mental health during COVID-19 peaks, lockdown, and times of curfew, many studies have used the LPA/LCA person-centered approach to uncover and explore unobserved groups. However, the majority of research has focused only on negative psychological concepts to explain mental health. In this paper, we take another perspective to explore mental health. In addition, the study focuses on a period of peak decline in the COVID-19 pandemic.

**Objective:**

The present paper aim (a) empirically identifies different profiles among a cohort of Facebook users in Tunisia based on positive factors of mental health using a person-centered approach, (b) outline identified profiles across sociodemographic, internet use, and physical activity, and (c) establish predictors of these profiles.

**Methods:**

Cross-sectional data were collected through an online survey among 950 Facebook users were female (*n* = 499; 52.53%) and male (*n* = 451; 47.47) with an average age =31.30 ± 9.42. Subjects filled *Arabic version of Satisfaction with Life Scale, Scale of Happiness (SWLS), Gratitude Questionnaire (GQ-6), International Physical Activity Questionnaire (IPAQ), and the Spirituel Well-Being Scale (SWBS)*.

**Results:**

The LPA results revealed three clusters. The first cluster (*n* = 489, 51,47%) contains individuals who have low scores on the positive psychology scales. The second cluster (*n* = 357, 37,58%) contained individuals with moderate positive psychology scores. However, a third cluster (*n* = 104, 10,95%) had high positive psychology scores. The selected variables in the model were put to a comparison test to ensure that the classification solution was adequate. Subsequently, the clusters were compared for the variables of socio-demographics, use of the internet for entertainment and physical activity, the results showed significant differences for gender (low mental well-being for the female gender), socio-economic level (low for the low-income class), and physical activity (low mental well-being for the non-exerciser). However, no significant differences were found for the variables age, location, and use of the Internet for entertainment.

**Conclusion:**

Our results complement person-centered studies (LPA/LCA) related to the COVID-19 pandemic and can serve researchers and mental health practitioners in both diagnostic and intervention phases for the public. In addition, the GQ6 scale is a valid and reliable tool that can be administered to measure gratitude for culturally similar populations.

## Introduction

After the first case of the infectious disease COVID-19, discovered in Wuhan, China (December 2019) and the spread of a strain with many symptoms and causing high prevalence of hospitalization and/or death worldwide (1), unprecedented health emergency was imposed in several countries and a majority of public sectors were dramatically affected. In response to this health emergency, COVID-19 disease was declared as a pandemic labeled as “Public Health Emergency of International Concern (PHEIC)”, the World Health Organization (WHO) and Centers for Disease Control (CDC) began to respond to COVID-19 and its severe impact. Research in this context suggested that COVID-19 have been linked to several of the most significant health, social and economic troubles of the twenty-first century and 250 million people have tested positive for the virus since it began to spread in December 2019, and more than 5 million individuals have perished. Indeed, WHO has been found to have a broad range of physical health challenges and human behavior changes such as sedentary lifestyles, decreased physical activity, insomnia, mental health, disinformation, misinformation spread on the web and social networks, and problematic internet uses (2–4). Furthermore, fear of infection, frustration and boredom, lack of supplies, worry of hospital overcrowding, and financial loss all contribute to the widespread emotional discomfort and increased risk of mental disorders associated with COVID-19 (5, 6), for example, more than a quarter of the Chinese society reported some degree of psychological distress during the first wave of COVID-19 (7). Similarly, other disorders were revealed after the onset of symptoms such as fever, tiredness, and prolonged dry coughs (8), as were social avoidance, anxiety, concern of illness, and global panic (9). Likewise, security guidelines have forced governments to take precautions that ensure physical distancing and self-isolation, such as closing schools, universities, recreational parks, quarantine and firewalls (10, 11). These measures have influenced the quality of life of the majority of people and have resulted in a systematic negative impact on public mental health (12). Several studies have reported unusual and alarming levels of stress, anxiety and depression (13). There has also been an increase in loneliness, self-harm, and suicidal thoughts (14, 15). While high mortality rates have been noted among vulnerable groups (the elderly, obese, diabetics, hypertensive, etc…), the negative effects of the pandemic of the general public's mental health and wellness challenges have been published and well documented in different populations through online collected data (16–18). The majority of studies have agreed that the pandemic has a devastating strategic effect on the deterioration of the health care system which has already been observed in several countries (19–23). However, studies in human psychology and public health in the pandemic context have focused primarily on mental disorders [for example, (13, 24, 25)].

Little research has ranked individuals based on their positive mental health (26). Despite the role of positive psychology factors in the prevention of mental health problems (27), a recent meta-analysis involving internet users reported a trend toward negative mental health parameters such as depression, anxiety, suicidal ideation, fear and stress (28).

Moreover, most of these studies have not given importance to the social and religious context. In fact, religious involvement has been identified as protective factors for mental health (29, 30) and stimulating the positive psychology factors. As an example, spiritual well-being has been highlighted as a key element of social resilience during times of crisis (31, 32). Also, gratitude as a highly valued moral affect in religions (33, 34), was associated with psychological well-being and satisfaction with life (35).

During the COVID-19 pandemic, two mixture modeling techniques have been widely used to segment groups based on several psychological concepts of mental health. The first is Latent Class Analysis (LCA) which deals with qualitative variables, and the second is Latent Profile Analysis (LPA) which deals with continuous variables (36). Latent Profile Analysis (LPA) is a flexible, model-based clustering procedure that supports the probabilistic identification of mental health subgroups. Using this technique, several mental health clusters have been identified for the general population in different countries based on psychopathological symptoms (e.g., stress, anxiety, and depression). But to our knowledge, no study has applied this procedure to class cohorts among positive psychological.

Due to the spread of health-related misinformation and disinformation on social media in problematic ways (37, 38), it is very interesting to target vulnerable groups like Facebook users. Indeed, the massive dissemination of disinformation on the web and social media platforms negatively effects on mental health [see for example: (38)]. In addition, phenomena of Internet addiction have been reported (39). Tunisia can be a favorable geographical space for these problems. The pandemic in this country was associated with high mortality rates (40, 41), behavioral changes (42) and mental health problems (43). Correspondingly, serious internet addiction problems have been reported (41). Furthermore, the country had 6.5 million Facebook users as of January 2020, which is equivalent to 55 percent of the country's total population. As an example, in pandemic, Sediri et al. (44) found that adult Tunisian women were suffering from severe depression, anxiety, and stress symptoms. Women's use of social media was found to be problematic in ~40% of cases.

Therefore, the objectives of this study are: (a) empirically identifying, from positive factors of mental health, different profiles among a cohort of Facebook users in Tunisia based on person-centered approach, (b) to outline identified profiles, across the sociodemographic, internet use and physical activity and (c) establish predictors of these profiles.

## Methods

### Data Collection and Procedures

Cross-sectional data were collected through a survey designed online using the Google Forms application from October 04 to 28, 2021. We used a snowball sampling method to collect information from Tunisian Facebook users to circulate the questionnaire and involve the maximum number of target people. This method is increasingly applied in studies involving social network users (45, 46). Initially, invitations to fill in an informed consent by specific Google Gmail accounts were distributed on several groups of the social network Facebook. Subsequently, the respondents invited their friends to complete the survey. This procedure makes it possible to create a specific ballot box, in order to be able to control multiple responses. We used this environment based on the Google application's Cloud Computing system which allows for a single response per user. However, the use of this algorithm requires having a Google E-mail address and prohibits access to Internet Protocol (IP) addresses of users for reasons of confidentiality, privacy and security. In the response form, no personal information was obtained (e.g., names, home addresses, email addresses, and phone numbers). While the study follows the Recommended Standards for Conducting and Reporting Online Surveys “CHERRIES” (47).

The inclusion criteria concern each Facebook user aged 18 and over, residing in Tunisia and whose mother tongue is Arabic. However, subjects who do not reside in the country are excluded from the study to maintain the same social and cultural context at the time of the survey.

The study was approved by the local ethics committee of the Institute of Sport and Physical Education of Kef, Jendouba University in Tunisia.

According to Weber et al. (48) the number of Facebook users in Tunisia was 6.5 million. We used Raosoft online sample size calculator (49) and formulas to define subjects needs for this online survey. The method of sampling used in similar previous studies suggested a sample size of 664 as a minimal appropriate participant by assuming a 66% percent response rate, 5% precision or margin of error, and 50% proportion with a 99% confidence interval.

The number of questionnaires was 1,023 regular internet users. We used Mahalanobis distance to eliminate the questionnaires with outlets responses for example random responses and psychological problematic cases (*n* = 73), 950 copies of the measurement instrument were retained. While 8.11% (*n* = 77) of these participants reported having been ill with coronavirus at some point during the pandemic. Participants were female (*n* = 499; 52.53%) and male (*n* = 451; 47.47%) with an average age =31.30 ± 9.42 years. All subjects were of Muslim religion and had permanent access to the internet.

The details of the socio-demographics of the participants and their distributions according to the variables are presented in **Table 2**.

## Instruments

### Sociodemographic Questionnaire

The information solicited on the socio-demographic variables was of age, gender, nationality, country of residence, religion, education level was binary coded (0 < higher; 1 = higher), their residence status (0 < rural; 1 = urban), family income (coded low; medium and high). In addition, access to the internet and its use as a means of entertainment was binary coded (0 = no; 1 = yes).

#### Arabic Satisfaction With Life Scale [ASWLS]

Among the primary measures of interest in this study was the Satisfaction of Life Scale (SWLS) (50, 51). According to Google Scholar statistics from November 2021, this scale was mentioned in 32,791 papers. This statistic alone demonstrates the magnitude of its impact on the world of study (52). A five-item Likert-type scale has excellent psychometric qualities in terms of both reliability and validity. In terms of reliability, its internal consistency often runs between 0.79 and 0.89, and its rank in item-total correlations typically ranges between 0.51 and 0.80 (53). Indices have been observed to oscillate between 0.83 for 1-month intervals (54), 0.83 for 2-month periods (51), and 0.54 for 4-year periods (53). Regarding the factorial invariance, distinctions in sex or age are seldom seen.

#### Arabic Scale of Happiness [ASH]

In Arabic context, there are just a few happiness measures. The scale of happiness included 15 short statements as well as five-filler items. Each item was graded on a five-point scale of intensity. The overall score can vary between 15 and 75, with higher numbers indicating greater satisfaction. The results of a primary axis factor analysis, followed by oblique rotation (pattern and structural matrices), provided two factors: general happiness and successful life. Correlations between items and the remainder of the exam varied from 0.42 to 0.77. Internal consistency and temporal stability were shown by Cronbach's alphas and test-retest reliability ranging from 0.82 to 0.94. The Arabic Scale of Happiness (55) had statistically significant correlations with mental health, life satisfaction, optimism, love of life, and self-esteem, demonstrating construct validity (55). Male college and high school students scored higher than their female counterparts did on average. Male and female undergraduates scored higher than their teenage counterparts did on average. The Arabic Scale of Happiness was shown to have strong psychometric qualities. For the present study, we use an average of the total score of the instrument.

#### Gratitude Questionnaire [GQ-6]

The GQ-6 is a six-item questionnaire designed to assess the dispositional element of gratitude (56). Each item is graded on a seven-point scale ranging from one (strongly disagree) to seven (strongly agree). A simple item is “I have so much to be thankful for”. After reversing pertinent items, the scale scores are the total of the items. The scale's higher scores indicate a stronger sense of gratitude. The scale was translated into Arabic using a forward-backward translation process for the purposes of this study. The GQ-6 has strong psychometric qualities in the original article, with a solid one-factor solution and high internal consistency. The internal consistency reliability of the six-item scale, measured by the Cronbach's alpha, was 0.82.

#### The International Physical Activity Questionnaire (IPAQ)

IPAQ have two available versions: long (five activity domains asked separately) and short (four general items), which may be used through telephone or self-administered techniques. The surveys' goal is to provide standardized instruments that may be used to collect data on health-related physical activity that can be compared across borders. The development of an international physical activity measure began in Geneva in 1998, and extensive reliability and validity testing was carried out across 12 nations (14 locations) in 2000. The final findings indicate that these measures have acceptable measuring qualities for applications in a variety of countries and languages, and that they are appropriate for national population-based prevalence investigations of physical activity participation (57).

In the present study, the Arabic version of the (IPAQ-S) was used. The scale exhibits robust psychometric properties in terms of reliability and validity (58).

#### The Arabic Version of the Spiritual Well-Being Scale [SWBS]

The Spiritual Well-Being Scale (SWBS) was developed over 30 years ago (59, 60) and has since become a widely used and well-researched tool (61). Despite the fact that the SWBS was initially established in a Christian context and influenced by the Judeo-Christian idea of well-being, Ellison (59) claimed that it is a nonsectarian tool that may be used by other religions that have a personal experience of God. As a result, the SWBS was produced to be extensively used to assess spiritual well-being in religious and unreligious people, as well as people of other religions and cultures.

The SWBS is a self-report paper–pencil instrument with 20 items. It takes 10–15 min to finish. On a six-point Likert scale, ranging from strongly agree to strongly disagree, each item is answered. The RWB and EWB subscales are the two subscales of the SWBS. Ten items are intended to assess RWB and include the term “God,” whereas ten items assess EWB and include questions on life fulfillment and direction. To reduce any potential response bias, around half of the items are written in the other manner. Each SWBS item is scored on a scale of one to six, with a higher number indicating greater well-being. Negatively worded items are recorded in the reverse way.

The SWBS and its subscales have great internal consistency, according to the reliability results. Cronbach's alpha scores for the SWBS climbed from 0.66 to 0.85. Cronbach's alpha values for the Arabic SWBS (62) and its subscales (RWB and EWB) were similar to those of other studies with varied samples using the original English version of the SWBS (59, 63, 64), who demonstrated that the SWBS has good internal consistency and reliability consistency. Overall, the SWBS and its subscales are valid and reliable measures that may be used with the population in the Arabic Islamic culture.

### Statistical Analysis

Statistical analyses and confirmatory factor analyses were performed using SPSS Version 26.0.0.0 (IBM, USA) and SPSS Amos software Version 23.0.0.0 (IBM, USA) respectively. While the Mclust and Tidy LPA R Studio packages have been adopted for LPA.

The preliminary data analysis was performed by Skewness and Kurtosis normality tests. First, scores for the adapted scale GQ6 were undergone exploratory factor analysis, which performed by the Unweighted Least Squares method with Promax rotation and Kaiser-Mayer-Oklins (KMO) normalization. We retained solutions for KMO > 0.60, Eigenvalue > 1 and a significant Bartlett test (Chi2). The GS6 structure was inspected by confirmatory factor analysis (CFA). Several adjustment indexes of the CFA were retained to examine the model: (1), (2) Goodness of Fit Index GFI. (3) Adjusted Goodness of Fit Index AGFI; (4) Comparative Fit Index (CFI); (5) the Tucker-Lewis index (TLI); (6) Root mean square residual (RMR) and (7) the Root Mean Square Error of Approximation (RMSEA). The χ2 must not be significant; however, this criterion is very criticized on large samples. While χ2/DF (DF = degrees of freedom) is widely used and must be less to 2 or superior to 5. According to the recommendations of Hu and Bentler (65), the GFI and AGFI must have values >0.90 to accept the model. TLI and CFI values >0.95 represent a good fit for the model. The RMSEA should be <0.06 for good model fit and <0.08 for acceptable model fit (65, 66).

The reliability of all positive psychology scales was achieved by calculating the internal consistency Cronbach's α coefficient. The recommended threshold for the indices is 0.70 to accept it and 0.80 for good reliability.

LPA were used to classify individuals (clusters) with similar characteristics in the various psychological tests performed. This approach is a well-known mixture-model for identifying homogenous latent classes or subgroups within a large heterogeneous group.

In this procedure, four Tidy LPA models (with 2, 3 and 4 classes) were investigated successively: model 1 (Varying means, equal variances, and covariances fixed to 0), model 2 (varying means, equal variances, and equal covariances), model 3 (Varying means, varying variances, and covariances fixed to 0) and model 6 (Varying means, varying variances, and varying covariances). Before analysis, a robust variant of the Mahalanobis distance based on the minimum covariance determinant was considered to detect and delete multivariate outliers.

The fit of the latent profile model is assessed using a variety of statistical measures. (1) Bayesian information criterion [BIC; (67)]. According to several studies (68, 69), this is the most reliable indication of model fit. The BIC encourages models to be as simple as possible, and it can be used to compare competing LPA solutions. BICs with a lower value suggest a better fit. (2) Akaike's information criterion was being studied (AIC). Similarly, a significant value of the bootstrap likelihood ratio test (BLRT) was also considered in selecting the number of classes. (3) The BLRT uses a Bootstrap resampling method to approximate the *p*-value of the generalized likelihood ratio test. (4) Entropy values that are equal to or >0.80 are associated with 90% accurate assignment accuracy, while entropy values of 0.64 and below are associated with high classification error rates.

The comparison between the clusters on all the variables of the LPA model was carried out by the Multivariate analysis of covariance (MANCOVA).

The comparison between clusters of each continuous variable was performed by one-way variance analyses with Bonferroni post-hoc test. In addition, Effect size (Eta Squared) was examined for each comparison. While categorical variables comparisons were made by Chi2 tests with Cramer's *V* effect size.

Completely, gender, family income, academic level, dwelling, and physical activity practice were used in a multinomial logistic regression analysis (with age as a Covariate) to see whether factors had a significant impact on positive mental health outcomes.

## Results

At first, the data was visually inspected to make sure that there were no anomalies in the cases, then the skewness and kurtosis coefficients. Scale scores did not present any problems of normality (see Table 1).

**Table 1:** Latent profile fit statistics for attribute preference model with four models and five profiles.

**Model**	**Classes**	**AIC**	**BIC**	**Entropy**	**prob_min**	**prob_max**	**BLRT_p**
1	2	10,188.05	10,280.32	0.94	0.98	0.99	0.01
1	3	8,638.08	8,764.35	0.94	0.96	0.98	0.01
1	4	8,051.22	8,211.48	0.90	0.91	0.99	0.01
2	2	9,960.84	10,082.26	0.92	0.97	0.98	0.01
2	3	8,570.54	8,755.09	0.94	0.96	0.98	0.01
2	4	7,884.34	8,132.02	0.92	0.92	0.98	0.01
3	2	7,590.89	7,756.01	0.76	0.90	0.95	0.01
**3**	**3**	**7,414.30**	**7,613.42**	**0.83**	**0.90**	**0.94**	**0.01**
3	4	7,376.37	7,609.48	0.75	0.75	0.95	0.01
6	2	7,371.83	7,638.94	0.70	0.90	0.92	0.01
6	3	7,225.43	7,628.51	0.80	0.90	0.92	0.01
6	4	7,099.44	7,638.50	0.72	0.78	0.95	0.01

*Bold values: retained model*.

Before entering the scores of the scales in the LPA model, we carried out a psychometric examination for the GQ-6 since the scale has not been validated on an Arab population. In addition, a check of the internal consistency of the factors of the other scales was carried out to ensure that our data are adequate for the analysis.

We psychometrically tested the adapted version of GQ-6 through exploratory factor analysis, examination of its reliability through Cronbach's alpha internal consistency measure and confirmatory factor analysis.

The Kaiser-Meyer-Olkin index for the sampling quality measure was 0.90 with Bartlett Chi-square = (2,799.70, ddl = 15; *p* < 0.01) sphericity test value. The univariate one-factor model explained 64.31% of the total variance (Eigen value = 3.86).

The results of the confirmatory factor analysis provided a Chi2 value = 46.86 (ddl = 12; *p* < 0.01) with indices (AGFI = 0.96; GFI = 0.98), (CFI = 0.99; TLI = 0.98 and for the measurement error RMR = 0.03; RMSE = 0.067 90 % CI [0.049–0.086].

Subsequently the reliability of the other scales was examined by the same internal consistency coefficients. The results confirmed the reliability of the measurement scales. Indeed, for GQ-6, the coefficient alpha was 0.89 (95%CI [0.88–0.90]).

For spiritual well-being scale alpha = 0.86 (95%CI [0.85–0.088]) and 0.87 (95%CI [0.86–0.088]) for SWB and EWB, respectively.

Similarly, alpha values were = 0.88 (95%CI [0.87–0.90]), alpha = 0.85 (95%CI [0.84–0.86]), alpha = 0.88 (95%CI [0.87–0.90]) For SWLS, AHS and FS.

All four models were examined for 2- to 4-class solutions. The lowest Aic and BIC values were highlighted for model 3 (Aic = 7,414.30; Bic = 7,613.42) and model 6(Aic = 7,225.43; Bic = 7,628.5). Examination of these two indices gives us results that are favorable to the three-class model 6, since the two entropies for 4 clusters are 0.75 (model 3) and 0.72 (model 6), respectively. Also, the posterior probabilities of cluster membership for affected individuals are in the range [0.90–0.94] and exceeded a minimum threshold of 0.70.

The model fit indices from the latent profile analysis are presented in Table 1. Among the four models tested, the model, which presents the most values of Aic and Bic and an adequate entropy, is model 3.

**Figure 1:**
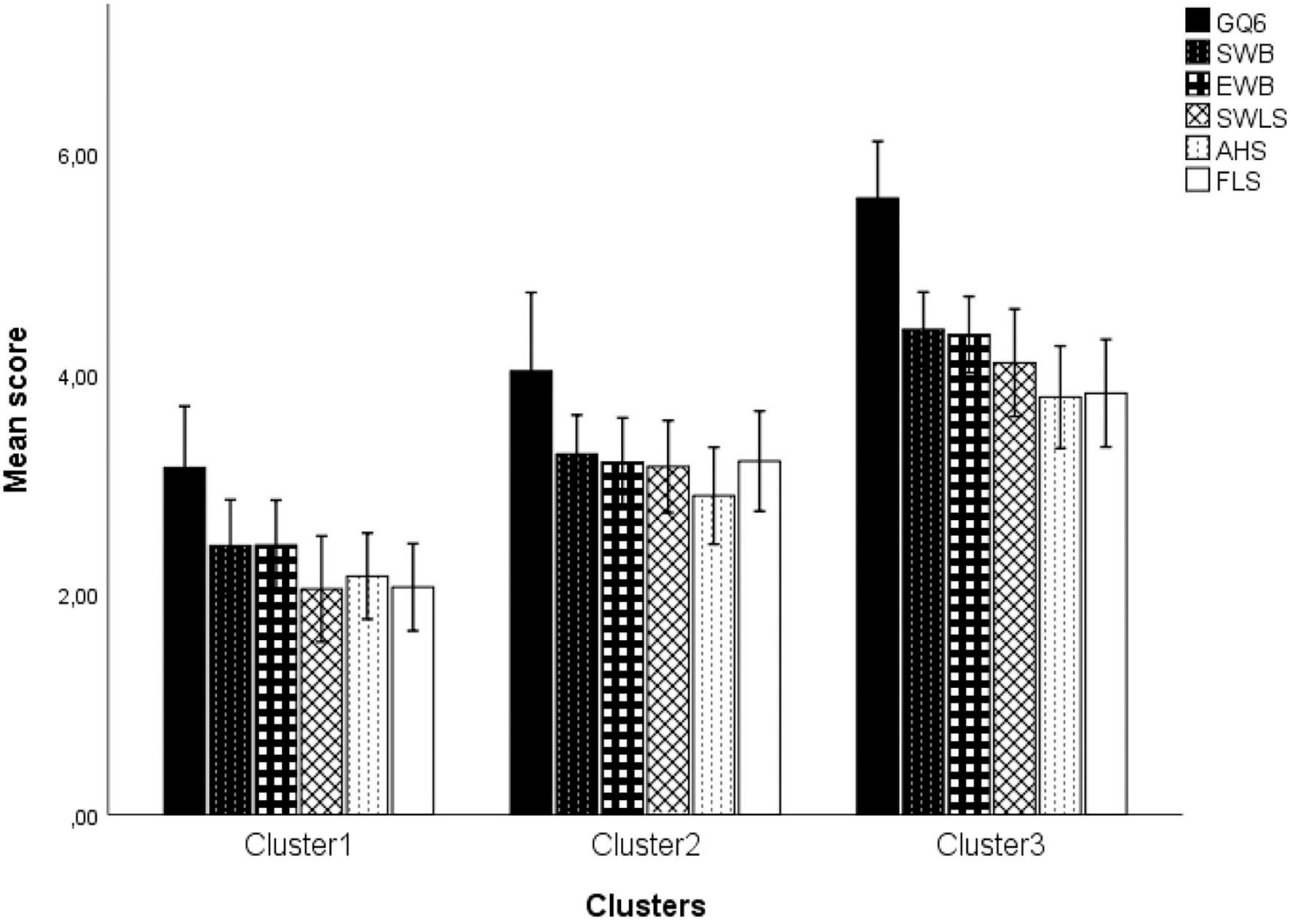
Distribution of the mean scores of scales according to the cluster.

To ensure the robustness of the solution, an analysis of variance tests with the scores of the five scales was performed. On all the scales, very significant differences were demonstrated (*p* < 0.001). In addition, the Bonferroni Post-Hoc test showed that cluster 3 has the highest scores on all positive psychology scales, cluster 2 has the moderate scores and cluster 1 has the lowest scores (see Figure 1).

As shown in Table 2, the first cluster is formed by 59% women, 40.90% men with a mean age of 31.07 ± 9.46. This group is divided into 37.63% with low family income, 40.08% with middle income and 22.29% with high family income. The academic background of this group of people was mostly higher education (62.78%) and almost 37% reported that reported that they use Internet as entertainment medium. According to the practice of physical activity, the distribution of individuals was low (37.83%), average (39.06%) and vigorous (23.11%).

**Table 2:** Characteristics of the three clusters.

**Variables**		**Clusters**			**Chi2/*F* Value**	**Cramer's *V***
			**Cluster1**	**Cluster2**	**Cluster3**		
Gender	Female	*n*	289	174	36	23,91^**^	0,16
		%	59,10%	48,74%	34,62%		
	Male	*n*	200	183	68		
		%	40,90%	51,26%	65,38%		
Socio economic level	Poor	*n*	184	94	29	27,81^**^	0,12
		%	37,63%	26,33%	27,88%		
	Medium	*n*	196	172	32		
		%	40,08%	48,18%	30,77%		
	High	*n*	109	91	43		
		%	22,29%	25,49%	41,35%		
Academic level	Graduate	*n*	307	218	71	1,79	0,043
		%	62,78%	61,06%	68,27%		
	Ungraduate	*n*	182	139	33		
		%	37,22%	38,94%	31,73%		
Dwellings	Urbain	*n*	306	243	70	2,98	0,06
		%	62,58%	68,07%	67,31%		
	Rural	*n*	183	114	34		
		%	37,42%	31,93%	32,69%		
Internet Entertainment medium	Yes	*n*	150	114	24	3,05	0,06
		%	30,67%	31,93%	23,08%		
	No	*n*	339	243	80		
		%	69,33%	68,07%	76,92%		
IPAQ	Weak	*n*	185	125	17	27,22^**^	0,12
		%	37,83%	35,01%	16,35%		
	Moderate	*n*	191	146	41		
		%	39,06%	40,90%	39,42%		
	Vigorous	*n*	113	86	46		
		%	23,11%	24,09%	44,23%		
Age			31,07 ± 9,46	31,10 ± 9,08	33,14 ± 10,20	2,231	0,005
GQ6			3,15 ± 0,56	4,03 ± 0,71	5,60 ± 0,52	732,872^***^	0,61
SWB			2,44 ± 0,42	3,28 ± 0,35	4,41 ± 0,34	1,285,721^***^	0,73
EWB			2,45 ± 0,41	3,20 ± 0,41	4,36 ± 0,35	1,092,456^***^	0,70
SWLS			2,05 ± 0,48	3,16 ± 0,42	4,11 ± 0,49	1,156,743^***^	0,71
AHS			2,17 ± 0,39	2,90 ± 0,44	3,79 ± 0,46	770,985^***^	0,62
FLS			2,07 ± 0,40	3,21 ± 0,46	3,83 ± 0,49	1,139,933^***^	0,71

Overall MANCOVA: Wilks' Lambda =0.38; F (6, 943) = 95.86*** (**Eta** =0.62).

** *: p < 0.01*;

*** *: p < 0.001*.

*The first profile (51.47%) presents vulnerable cluster in terms of positive mental health. The second profile (37.58%) presents clusters with moderate positive mental health. The third profile (10.95%) presents people in good positive mental health*.

While the second cluster is composed of 48.74% women and 51.26% men with a mean age of 31.10 ± 9.08. This cluster is subdivided for the family income variable: into low (26.33%), medium (48.18%) and high (41.35%). Nearly 61% of this group had higher education. In addition, 31.93% of the individuals reported that they use Internet as entertainment medium. The examination of physical activity in this group showed the following results: 35.01% are physically inactive, 40.90% are moderately active and 24.09% practice vigorous physical activity (see Table 2).

The third cluster contains 34.62% of women and 65.38% of men with a mean age of 31.10 ± 9.08. The repair by family income for cluster 3 was 27.88% for low levels, 30.77% for medium level and 41.35% for high levels. 23.08% of individuals in this cluster reported that they use Internet as entertainment medium. The majority of this group performs rigorous physical activity (44.23%), compared to 39.42% who perform moderate physical activity and 16.35% who are physically inactive (Table 2).

No significant difference between the three clusters was demonstrated for the place of residence (urban vs. rural) and the Internet entertainment medium.

### Multinomial Logistic Regression

Modeling the likelihood of predicting class memberships was done using multinomial logistic regression models. The calculated standard error (SE), Wald test values, and adjusted odds ratio (AOR) with their 95 percent confidence intervals are summarized in Table 3.

**Table 3:** Multinomial logistic regression for the positive mental health profiles.

**Clusters^£^**	**Predictors**	**SE**	**Wald test**	**AOR**	**95% Confidence Interval for AOR**	
					**Lower Bound**	**Upper Bound**
Cluster1	Age^a^	0.01	2.66	0.98	0.96	1.00
	[Gender^b^=Female]	0.25	20.59	3.05^***^	1.88	4.94
	[Family Income^c^=Poor]	0.28	7.09	2.11^**^	1.22	3.67
	[Family Income =Medium]	0.27	7.92	2.16^**^	1.26	3.70
	[Academic level^d^=Graduate]	0.26	3.69	0.61	0.37	1.01
	[Dwelling^e^=Urban]	0.24	0.42	0.86	0.53	1.37
	[Internet^f^=No]	0.26	0.90	0.78	0.47	1.31
	[IPAQ^g^ =Weak]	0.32	14.64	3.38^***^	1.81	6.31
	[IPAQ=Moderate]	0.26	2.82	1.55	0.93	2.57
Cluster2	Age^a^	0.01	2.86	0.98	0.96	1.00
	[Gender^b^=Female]	0.25	7.28	1.97^**^	1.20	3.22
	[Family income^c^=Poor]	0.29	0.89	1.32	0.74	2.33
	[Family income=Medium]	0.28	8.97	2.29^**^	1.33	3.94
	[Academic level^d^=Graduate]	0.26	2.68	0.65	0.39	1.09
	[Dwelling^e^=Urban]	0.25	0.23	1.13	0.69	1.83
	[Internet^f^=No]	0.27	1.46	0.72	0.43	1.22
	[IPAQ^g^ =Weak]	0.33	12.63	3.18^***^	1.68	6.01
	[IPAQ=Moderate]	0.27	2.54	1.53	0.91	2.57

£ *Class 3, reference*;

*SE, standard error; AOR, Adjusted Odds Ratio*;

a *age, Covariate*;

b *male, reference*;

c *High Family Income, reference*;

d *ungraduated, reference*;

e *Rural, reference*;

*Not uses internet for Entertainment, reference; Vigorous, reference*.

** *p < 0.01*;

*** *p < 0.001*.

Results of multinomial logistic regression analysis indicated that poor mental health class were related to female gender (AOR = 3.05; 95% CI: 1.88–4.94), poor economic level (AOR = 2.11; 95% CI: 1.22–3.67), medium Family Income (AOR = 2.16; 95% CI: 1.26–3.70), and weak physical activity (AOR = 3.38; 95% CI: 1.81–6.31). However, good mental health was associated to gender (AOR = 1.97; 95% CI: 1.20–3.22), medium Family Income (AOR = 2.29; 95% CI: 1.33–3.94) and Weak physical activity (AOR = 3.18, 95% CI: 1.68–6.01) (see Table 3).

## Discussion

The present paper aim (a) empirically identifies different profiles among a cohort of Facebook users in Tunisia based on positive factors of mental health using a person-centered approach, (b) outline identified profiles across sociodemographic, internet use, and physical activity, and (c) establish predictors of these profiles.

Initially, an adaptation of the GQ-6 scale was required to measure gratitude. The initial version of the instrument underwent translation into Arabic using the committee method and was subjected to both exploratory and confirmatory factor analysis to test its structure. The results of both analyses confirmed the uni-factorial model initially established. Adaptations of the gratitude questionnaire (GQ-6) in Brazil support our evidence of the validity and reliability of the scale for a single-factor structure (70). The study confirmed a unidimensional solution for two different samples (CFI = 0.99 and CFI = 0.97) with Cronbach's alpha of 0.87. However, the study of Dixit and Sinha (71) kept the same factor structure, but with only five scale items with an alpha reliability of 0.74.

Before proceeding to the identification of the profiles, reliability tests by calculating the classical Cronbach's alpha coefficient with confidence intervals on all the scales was carried out to ensure the reliability of the measures. The results were satisfactory and made it possible to integrate all the scales into an LPA model since all the scales presented an adequate internal consistency.

The LPA results revealed three clusters. The first cluster contains individuals who have low scores on the positive psychology scales. The second cluster contained individuals with moderate positive psychology scores. However, a third cluster had highly positive psychology scores. The selected variables in the model were put to a comparison test to ensure that the classification solution was adequate. Subsequently, the clusters were compared to the variables of socio-demographics, use of the internet for entertainment and physical activity, the results showed significant differences for gender (low mental well-being for the female gender), socio-economic level (low for the low-income class), and physical activity (low mental well-being for the non-exerciser). However, no significant differences were found in the variables age, location, and use of the Internet for entertainment.

According to the findings of a multinomial logistic regression study, poor mental health was linked to female gender, low economic status, medium economic status, and low physical activity. On the other hand, good mental health was related to gender, a middle socioeconomic status, and a lack of physical exercise.

To our modest knowledge, no studies have attempted to identify latent groups (LPA or latent class analysis on categorical variables LCA) from positive psychology parameters in the context of COVID-19. However, several studies from a negative or mixed (negative/positive) perspective has been highlighted profile identification for psychological distress, well-being and general mental health from online surveys. As an example, Pierce et al. (72) used LPA techniques to identify psychological distress clusters based on symptoms using the Brief-Symptom Inventory-53. Three latent classes defined by the level of symptom severity were identified (mild, moderate, and severe). Similarly, in another study incorporating negative mental health constructs, Fernández et al. (73), tested an LPA model at ~4,400 subjects in Argentina that used the constructs of distress and anxiety. Following the analysis, the classification resulted in three profiles that justified the model. However, the results were related to the quarantine phase. In another study, Yalçin et al. (74) identified three latent profiles among University students in Turkey from fear, depression, anxiety, stress, mindfulness, and resilience related to COVID-19. The results also revealed that 38% of the participants were classified in the low psychological symptoms profile vs., 16% who were classified in the high psychological symptoms group. Similarly, female gender was related to high symptoms.

In another example, Fernandez-Rio et al. (75) identified three groups of mental well-being: high (with low depressive symptoms, higher effect and resilience), moderate, and low for an age range above 16 years. In line with the present study, similar results were put for physical activity and gender variable. In fact, the group that presented a highly mental well-being practiced a vigorous and moderate physical activity before the quarantine (81.1%), in addition it contains much fewer women. Similarly for the gender variable, previous research (76–78), indicates that the female gender has a significantly higher risk of psychosomatic health problems and low life satisfaction compared to boys. Fischer (79) explains girls' low mental well-being as a result of being expected to be more emotionally sensitive and expressive.

Regarding the practice of physical activity, the current results agree with a paper by Zhang and Chen (80) highlighted a positive correlation between physical activity, Happiness, and life satisfaction, which are two components of Chinese students' subjective well-being.

Consistent with our study for the family income variable, (81), in a survey of health and well-being for students in Wales, UK, showed that latent classes with higher mental well-being were more affluent. Also, other studies have established strong links between economic standard of living and mental well-being, however other results have suggested the presence of mediating variables, for example the feeling of insecurity among workers (82).

However, our results were not able to show differences between classes according to age, on the other hand, the study of Bernabe-Valero et al. (83) found an inverse association between negative effect and age, indicating that the higher the age, the lower the negative affects scores. Other studies such as Bidzan-Bluma et al. (84) found that older individuals had better well-being scores than younger individuals. Within this framework, Ebert et al. (85), in a study with participants from the crowdsourcing platform, MTurk, found that mean age differences were observed. However, the trajectory of change did not differ by age. This suggests that responses to COVID-19 maybe age invariant and that effects on well-being are not immediate but may emerge over a longer period of time or in relation to social participation (86).

Daly et al. (87) reported different results for socio-demographic groups examined on mental health problems in a representative British sample. The increase was greatest among those aged 18–34, followed by women and those with higher incomes and education. However, the results that were reported at the beginning of the pandemic were variable over time.

Regarding the association between Internet use and mental health, previous studies have discovered mixed results and depend on several factors. For example, Lam et al. (88) found that frequent Internet use might have beneficial effects on depression and life satisfaction in older adults.

From a different angle, the found clusters point to strong links between thankfulness and spiritual well-being and the other positive psychology variables. Several research (89–92) have shown correlations between religion, well-being, stress management, and happiness. Many additional studies have also shown a link between spirituality and dimensions of subjective well-being including life satisfaction, optimism, self-esteem, and the sense of having lived a meaningful life (93–97). Spirituality may also help patients build psychological toughness and resilience, and patients who are conscious of their own inner strength can create positive attitudes (98, 99). Spirituality and religious coping behaviors (100, 101), such as prayer, supplication, Quranic recitation, trusting and remembering God, forgiveness, patience, starting the day with positive ideas, thanking God for His blessings, are likely to become a coping mechanism after a traumatic experience (32) and may be a key determinant of post-traumatic growth (102). During the pandemic, religious groups rallied to fight the epidemic and its ramifications, demonstrating that religion can have a substantial impact on communal perceptions in times of crisis (103). Spirituality, in this view, conveys hope for the future and may help people cope with problems (104). The COVID-19 pandemic, according to González Sanguino et al. (105), has raised persons' spiritual requirements has been reported to demonstrate the necessity of spirituality more clearly.

## Limitation of the Study

Similar to any research, this study had some limitations that we must point out.

First, the exploratory and confirmatory factor analysis of the GQ6 scale were conducted on a single sample and the discriminant and convergent validity were not examined. Future research should examine these psychometric tests across other samples.

Second, resilience as a specific mental health construct in the context of the pandemic has not been examined due to the multitude of scales used. It is crucial that it must be incorporated into other studies to complement our work. Specially, during this study, we did not examine pathological people in terms of mental health. Future research should consider this population.

Third, the study was cross-sectional, further longitudinal studies need to be conducted to examine the transition of latent profiles during different waves of the COVID-19 pandemic.

Finally, future research needs to examine the role of social media and changes in the quality of life and peer relationships that may help explain trends in mental well-being.

## Conclusion and Recommendations

The findings of the study led to the identification of three latent profiles: low, moderate, and high positive mental health. It has been shown that a large percentage of Facebook users are vulnerable in terms of mental health. The outcomes also revealed substantial gender, socio-economic, and physical activity practice differences. Moreover, the multinomial logistic regression analysis connected poor mental health to female gender, low socioeconomic position, middle socioeconomic status, and low physical activity. Mental health was linked to gender, middling socioeconomic class, and lack of physical activity.

This study, complement person-centered studies (LPA/LCA) related to the COVID-19 pandemic and can serve mental health researchers and practitioners in the diagnostic and intervention phase.

In addition, psychometric test results suggested that the Arabic version of the GQ-6 scale is a valid and reliable tool and can be administered to measure gratitude toward culturally similar populations.

A need to identify and analyze the constructs of positive psychology can inform the improvement of the practice of psychological intervention, prevention and improve social dialogue. Indeed, focusing on what is going well in life and the positive aspects can contribute to the optimal functioning and development of individuals.

Practical measures to manage our mental health during these difficult times include consuming official media and accessing reliable sources of information that can limit the spread of misinformation related to COVID-19.

## Data Availability Statement

The original contributions presented in the study are included in the article/Supplementary Material, further inquiries can be directed to the corresponding author/s.

## Ethics Statement

This work has received approval from the Ethics Committee of the “Research Unit, Sportive Performance, and Physical Rehabilitation, High Institute of Sports and Physical Education, Kef, University of Jendouba, Jendouba, Tunisia” and received ethical clearance from the UNESCO Chair “Health Anthropology Biosphere and Healing Systems,” University of Genoa, Genoa (Italy), the Higher Institute of Sport and Physical Education of Kef, Kef (Tunisia), and the Higher Institute of Sport and Physical Education of Sfax, Sfax (Tunisia). The proposal has been also approved by the Jendouba University Ethics Committee and was undertaken following the legal standards of the Helsinki declaration in 1964 and its corresponding amendments. The patients/participants provided their written informed consent to participate in this study.

## Author Contributions

NG, AT, NC, AF, NB, and RA have conceived and designed the experiment. NG, AT, NC, MS, JK, LP, AF, NB, and RA have collected and analyzed data. All authors drafted and critically revised the manuscript.

## Conflict of Interest

The authors declare that the research was conducted in the absence of any commercial or financial relationships that could be construed as a potential conflict of interest. The reviewer NR declared a shared affiliation with the author LP at the time of the review.

## Publisher's Note

All claims expressed in this article are solely those of the authors and do not necessarily represent those of their affiliated organizations, or those of the publisher, the editors and the reviewers. Any product that may be evaluated in this article, or claim that may be made by its manufacturer, is not guaranteed or endorsed by the publisher.
