# Supplementary material for: Latent Profile Analysis to Survey Positive Mental Health and Well-Being: A Pilot Investigation Insight Tunisian Facebook Users
Source: Front Psychiatry. 2022 Apr 7;13:824134. doi: 10.3389/fpsyt.2022.824134 (PMC9021554; doi:10.3389/fpsyt.2022.824134)
Supplement: Supplementary file 1 [file Image_1.pdf]

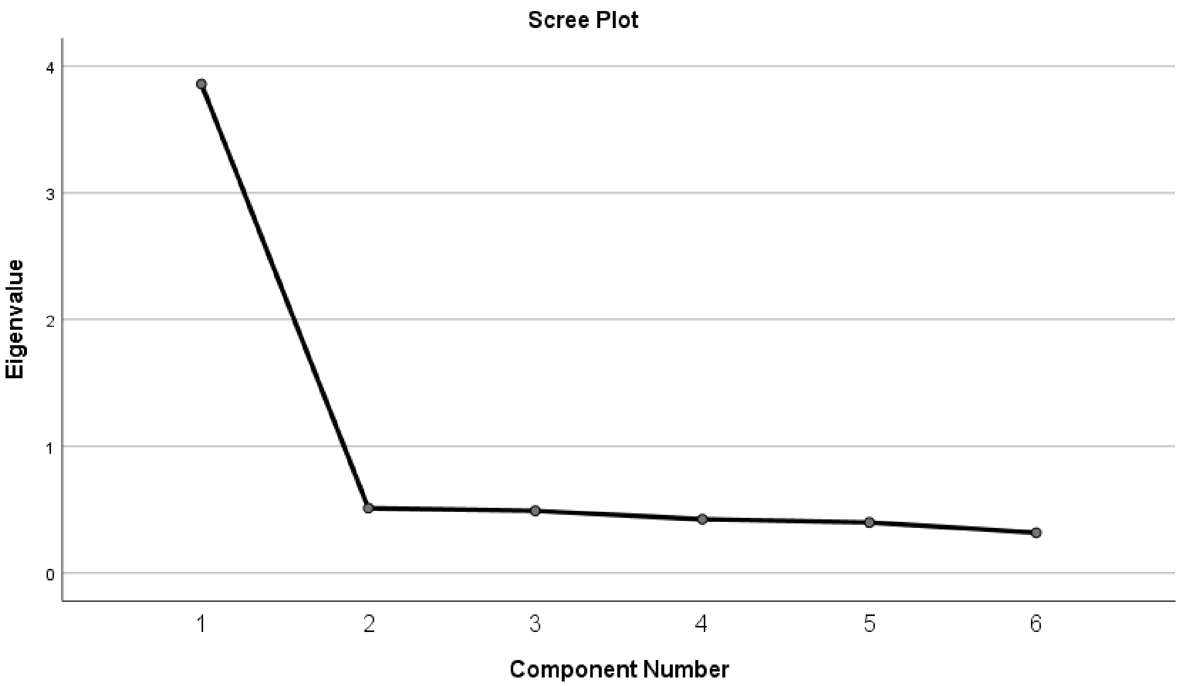

Figure 1. Scree plot of the GQ-6

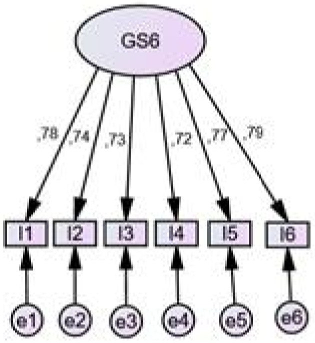

Figure 2. Confirmatory factor analysis of the GQ6. All parameters significant at p<0.01
